# Combining Soft- and Hard-Templating Approaches in MWW-Type Zeolites

**DOI:** 10.3390/molecules25153335

**Published:** 2020-07-23

**Authors:** Anderson Joel Schwanke, Jaíne Fernandes Gomes, Katia Bernardo-Gusmão, Sibele Pergher

**Affiliations:** 1 Universidade Federal do Rio Grande do Sul, Laboratório de Reatividade e Catálise, 91501-970 Porto Alegre Rio Grande do Sul, Brazil; anderson-js@live.com (A.J.S.); jaine_f_gomes@hotmail.com (J.F.G.); katia.bg@ufrgs.br (K.B.-G.); 2 Universidade Federal do Rio Grande do Norte, Laboratório de Peneiras Moleculares, 59078-970 Natal, Brazil

**Keywords:** soft-templating, hard-templating, MCM-22, MWW, 2D zeolites

## Abstract

A combination of hard-templating (HT) and soft-templating (ST) approaches was studied to obtain MWW-type materials with intermediate physicochemical properties. The HT methodology involved the introduction of carbon particles as hard templates during gel synthesis to obtain a layered zeolitic precursor (LZP) with particles possessing a microspherical morphology. The LZP obtained was treated with surfactants as soft templates to expand the layers of the LZP, followed by a pillaring procedure. The materials were characterized by X-ray diffraction, transmission and scanning electron microscopy, elemental analysis and N_2_ adsorption. The results demonstrate that the obtained material possesses intermediate properties from both approaches, with interparticle mesopores/macropores and pore sizes between 18 and 46 Å. However, the ST procedure causes a partial disruption of some microspheres, forming small crystallite aggregates, and results in a decrease in the number of interparticle mesopores/macropores previously formed by the HT method.

## 1. Introduction

Nanoporous materials are solids with porous features with molecular dimensions of around 100 nm; they can be used in a wide range of applications [1]. Within this class, zeolites are fascinating microporous crystalline materials that have been applied in different fields, especially in catalysis, adsorption, separation and fuel-cell technology [2,3]. These materials are built from SiO_4_ and AlO_4_^−^ tetrahedra (or other elements) that connect to each other via oxygen bridges [4]. Repetitive connections of these basic units form three-dimensional structures with large internal volumes due to the presence of uni-, bi- or tridimensional channel systems (interconnected or not) and cavities with molecular dimensions of up to 2 nm (microporous range) [5]. Thus, only molecules with adequate geometries can access the pore systems, leading these materials to be used as molecular sieves. According to the International Zeolite Association (IZA), 248 different framework topologies have been reported so far [6].

Among these 248 examples, some zeolites have a two-dimensional (2D) layered zeolitic precursor (LZP), which has a chemistry that is quite similar to that of other 2D materials, i.e., clays, graphite, layered double hydroxide, titanates and other metal oxides, because it is possible to obtain a range of swollen, hybrid organic–inorganic, pillared and delayered derivatives [7]. However, 2D zeolites offer even more features, such as high surface area, tunable acidity and microporous channels located in the proper layers. MWW zeolitic layers are a good example because each layer, with a thickness of 2.5 nm, has sinusoidal channels with diameters of 0.40 × 0.50 nm and hemicavities with diameters of 0.71 nm located on the upper and lower sides of each surface [8]. Two-dimensional LZPs are usually obtained after hydrothermal synthesis and avoid the calcination step for the removal of organic structure-directing agents (OSDAs). The most well-known example of an LZP features the MWW topology, and is known as MCM-22(P); its 3D derivative (after calcination) is called MCM-22 zeolite, which has bidirectional 0.40 × 0.55 nm channels formed by the condensation of silanol groups between the MWW layers [9].

The majority of zeolite modifications use bottom-up approaches involving soft-templating (ST) or hard-templating (HT) strategies [10]. The ST strategy uses dual templates, which act as an organic structure-directing agent (usually as a head group) and a long alkyl chain (such as a tail group) to prevent the growth and stacking of zeolitic structures along the c-axis while serving as mesoporogen agents [11,12]. Another ST example uses a cationic surfactant combined with swelling procedures in order to obtain expanded LZP derivatives, increasing the possibility of obtaining pillared or delaminated structures [13,14]. Swelling agents include alkyltrimethylammonium surfactants partially exchanged with OH^−^ ions or mixtures of surfactant salts and tetramethyl or tetraprophylammonium hydroxide based-solutions [15,16,17]. A high pH is required to separate the lamellae, which interact strongly via the hydrogen bonds between silanol groups located on the surface of each lamellae. Recently, the swelling of MWW colloids using simple surfactant salts, e.g., hexadecyltrimmethylammonium chloride, was reported [18].

On the other hand, the HT strategy uses larger particles derived from scaffolds, carbon, silica, polymers, starch-derived bread, carboxymethyl cellulose and bacteria, which are usually added to the synthesis gel and remain imbibed in the zeolitic crystals after crystallization; mesopores are then exposed after sacrifice by thermic or acid treatments [19,20,21,22,23,24]. In this way, meso-/macro- porous regions are created, facilitating the mass transport of bulky reactants into the active sites located in the micropores. Carbon particles with spherical, tubular, mesostructured and 3D-ordered macroporous structures are examples of hard templates used for the synthesis of MFI-type hierarchical zeolite [25,26,27,28]. However, HT approaches are relatively few in number for zeolites with MWW topologies [29]. Recently, we reported the synthesis of MWW zeolites with a HT methodology using carbon black pearls (BP 2000) via direct synthesis. However, in this case, the carbon particles did not stay imbibed in the zeolitic crystals; rather, they interacted with the external surface of the MWW LZPs, creating dandelion-like microspherical morphology of approx. 6–8 μm in size; additionally, interparticle meso-/macro- pores of between 50 and 3000 nm in size were formed by the random stacking of MWW crystallites [30].

To date, efforts to obtain MWW zeolites with mesoporous regions located between layers using ST methods and interparticle meso-/macro- pores by HT approaches have been studied from separate perspectives. Therefore, our objective was to obtain a unique MWW-type material with combined interlamelar mesopores formed after swelling and pillaring, and interparticle meso-/macro- pores with a dandelion-like microspherical morphology using carbon black pearls BP 2000. In this study, we were interested in exploring the effect of combining HT and ST methodologies for MWW-type zeolites and studying their physicochemical properties.

## 2. Results and Discussion

The synthesized materials were labelled according to the adopted experimental procedure. The material obtained after the synthesis of the standard LZP (without HT and ST) was labelled MWW(P). The material obtained after the synthesis of LZP using carbon black pearl as a hard template was labelled MWW(P)HT. The MWW(P)HT material was swollen with soft templates (surfactants) and the product was labelled MWW(P)HT-ST. Finally, the MWW(P)HT-ST material was pillared and calcined, and the product was named MWW-HT-ST-Pil. 

**Figure 1:**
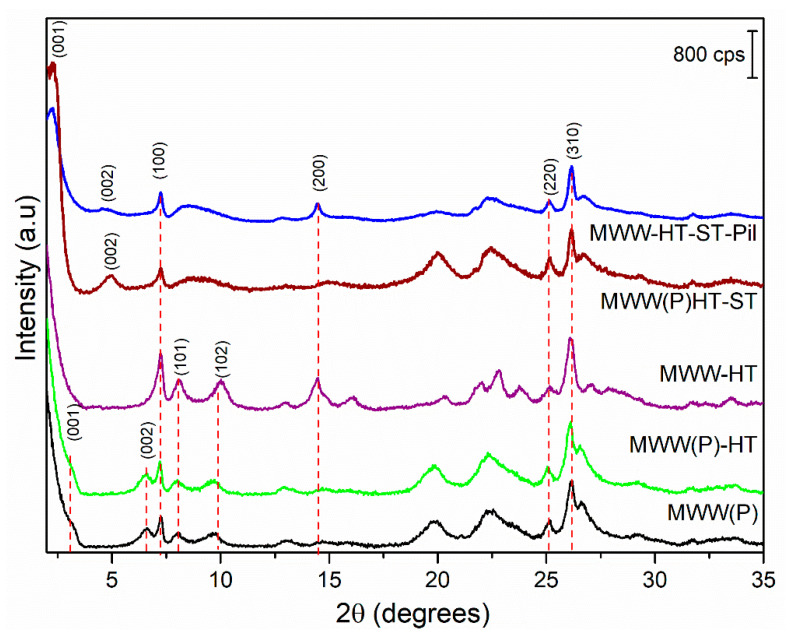
X-ray diffractograms of the MWW(P), MWW(P)-HT, MWW-HT, MWW(P)HT-ST and MWW-HT-ST-Pil zeolitic samples.

X-ray diffraction (XRD) diffractograms of the obtained samples are shown in Figure 1. All samples presented diffraction patterns which were characteristic of as-synthesized, swollen calcined, and pillared MWW-type zeolites [31]. The MWW(P)HT presented the same diffraction peaks as traditional MWW(P), and both as-synthesized precursors showed (002) reflection and a d_001_ spacing of 2.6 nm, which is typical of MWW-type LZPs. The MWW-HT (after calcination) material presented XRD peaks with stronger intensities, and the absence of (001) reflections suggests that tridimensional transformation had occurred. The swollen MWW(P)HT-ST sample presented widening of the (101) and (102) diffraction peaks, indicating the loss of layer stacking order along the c-axis. Moreover, the shifting of the (001) diffraction peak to lower angles indicated an increase in the d_001_ spacing to 3.9 nm, and confirmed the presence of surfactant cations between the interlayer regions. Therefore, the gallery regions (difference between the value of the d_001_ spacing, i.e., 3.9 nm, and the thickness of an individual MWW layer, i.e., 2.5 nm) were estimated to be 1.4 nm in height. Moreover, the presence of (002) reflections was evident, revealing a stacking of layers at a well-defined distance. The presence of (003) reflections was not observed due to the overlapping of (100) reflections. 

The XRD pattern of the pillared MWW-HT-ST-Pil sample featured a broadening of the (001) reflection, which confirmed some disturbance of the order of layers after calcination. However, the presence of (002) reflections was evident, indicating that the layers were stacked at a well-defined distance but with a random orientation. Moreover, the (001) reflections were maintained at the same Bragg angles as those of the swollen MWW(P)HT-ST, with a d_001_ spacing of 3.9 nm. This result confirmed that the silica pillars maintained MWW layers that were separated from each other.

The Si/Al molar ratios of the MWW(P) and MWW(P)-HT samples were found to be 23 and 22, respectively; these values are close to the theoretical gel molar composition. The Si/Al ratio of MWW(P)HT-ST was calculated to be 16, confirming the partial consumption of silicon from the zeolite layers due to the swelling treatment in the presence of caustic media. The AAS results for MWW-HT-ST-Pil showed an increase in the Si/Al ratio, i.e., to 37; this was due to the introduction of silica pillars.

**Figure 2:**
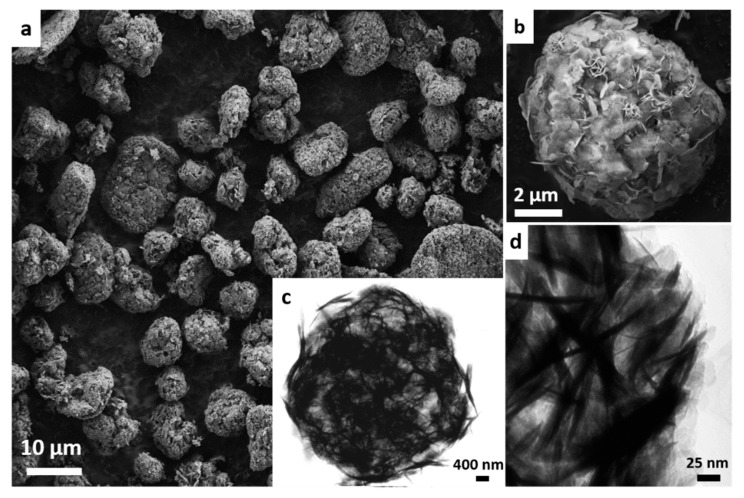
SEM micrographs (**a**,**b**) and TEM images (**c**,**d**) of the MWW-HT zeolitic product.

Figure 2 shows the micrographs of MWW-HT analyzed by scanning and transmission (c, d) electron microscopy (SEM and TEM, respectively). The SEM images (a) reveal that the majority of particles have microsphere morphologies with diameters between 5 and 8 μm, which is typical of a dandelion-like MWW zeolite [30]. Moreover, some particles possessed elongated and flat characteristics with sizes of approximately 12 μm and 2 μm, respectively. At higher magnification (image b), it was observed that the particles were formed by the aggregation of thin-flat crystallites with random orientation.

The TEM images (c,d) confirm the semihollow structure of the microspheres and the micro-/macro- voids obtained by the irregular orientation of the crystallites. We suggest that the main reason that the microspheres were still intact after calcination was because the MWW surface and edges were rich in silanol groups (Si-OH) which condensed with the silanol groups from other MWW crystallites with a random orientation to form silicon-oxygen-silicon bonds (Si-O-Si).

**Figure 3:**
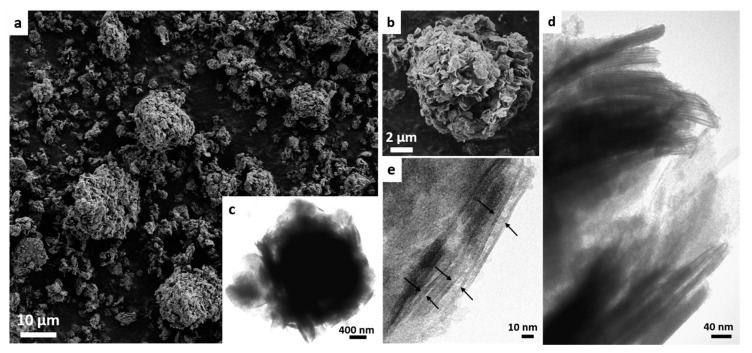
SEM micrographs (**a**,**b**) and TEM images (**c**–**e**) of the MWW-HT-ST-Pil product.

Figure 3 shows the morphology of the MWW-HT-ST-Pil sample. Some of the microspheres appeared to be intact, with the presence of many smaller particles, i.e., presenting sizes of approximately 2–3 μm. By comparing the micrographs of Figure 2a and Figure 3a, it is evident that the ST method caused a partial disruption of the microspheres and significant modification of their morphology in the final product. This effect may have occurred during the ST treatment, because the alkaline conditions could disrupt the Si-O-Si bonds that assembled the MWW crystallites to form silanol (Si-OH) and/or silanolate (Si-O^−^) groups. With higher magnification (image b), the microspheres were shown to possess a rougher surface, and their crystallites were more expanded than the thin flat crystallites shown in Figure 2b. This result is consistent with the increasing d_001_ spacing observed by XRD.

A TEM analysis of MWW-HT-ST-Pil (image c) indicated a decrease in the semihollow aspect of the microspheres, because regions with less density contrast were no longer observed, as shown in Figure 2c. However, swelling and pillaring increased the contribution of the interlayer expansion. This effect is also shown in Figure 3d, where a set of particles formed by the stacking of individual MWW crystals seems separated from each other by regions of less contrast density, and is clearly different to the flat elongated particles shown in Figure 2d. Figure 3e reveals some stacking of the set of elongated individual MWW crystals (thicknesses of 2.5 nm) separated from each other by regions with less contrast density.

**Figure 4:**
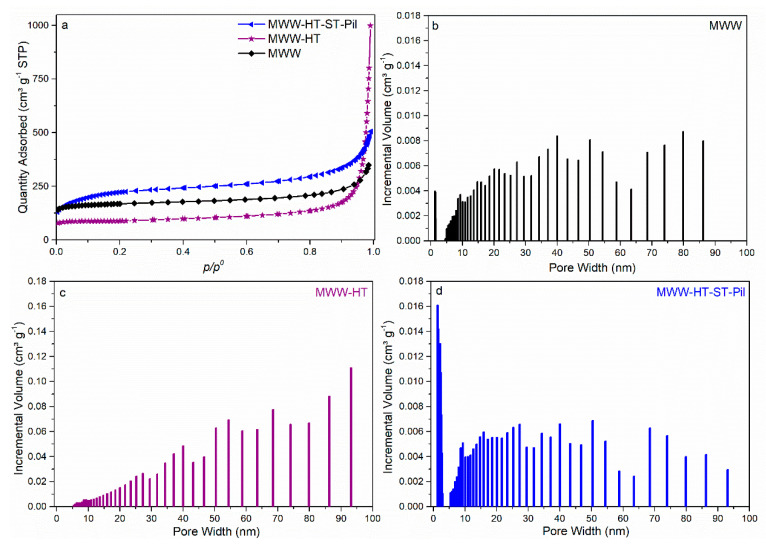
N_2_ adsorption isotherms (**a**) and DFT pore size distribution of the standard-MWW (**b**), MWW-HT (**c**) and MWW-HT-ST-Pil (**d**) zeolitic products.

N_2_ adsorption isotherms of MWW-HT-ST-Pil, MWW-HT and standard-MWW are shown in Figure 4. The standard-MWW zeolite presents type I isotherms at low *p/p*^0^ values and, according to the IUPAC recommendation, may be classified as a microporous-type material [32]. A moderate increase in N_2_ adsorption was also observed at *p/p*^0^ > 8, which indicated some contribution of interparticle meso-/macro- pores. The isotherm of MWW-HT also presented a type I profile at low *p/p*^0^ values, a moderate increase in the adsorbed N_2_ at *p/p*^0^ > 4 and a rapid increase at high volume pressures (near 1), indicating the presence of larger mesopores or narrow macropores, which was more pronounced than that of the standard-MWW material. Finally, the isotherm of the MWW-HT-ST-Pil material showed an increase in the amount of N_2_ adsorbed at low pressures, exhibiting strong adsorbate–adsorbent interactions followed by an adsorption step at *p/p*^0^ = 0.1–0.4, which is characteristic of capillary condensation that occurs in mesopores formed by pillaring. Moreover, the profile of the isotherm at *p/p*^0^ > 4 was more similar to that of standard-MWW than MWW-HT, suggesting a decrease in the number of interparticle meso-/macro- pores after the ST procedure. These results are consistent with the SEM analysis discussed above.

Figure 4 shows the pore size distributions of the synthesized samples. The standard-MWW sample exhibited a pore distribution between 5–85 nm (interparticle meso/macropores). The sample MWW-HT exhibited a wider pore distribution, i.e., between 5–136 nm, and a pore distribution maxima at 93 nm. These results indicate that the use of carbon black pearls generated MWW-type materials with a wider distribution of interparticle meso/macropores. These results are consistent with the TEM and SEM analyses, where the mesopores/macropore voids were more prominent in the MWW-HT sample. The sample MWW-HT-ST-Pil revealed a narrow pore distribution maxima, i.e., between 1.2–3.0 nm (supermicropores and small mesopores), by pillaring, and a wide distribution, i.e., between 5–94 nm, comprising the interparticle meso-/macro- pores. Therefore, the pore size distribution between the supermicropore regions, i.e., 1.2 nm, was consistent with the gallery height estimated by XRD, i.e., 1.4 nm. 

**Figure 5:**
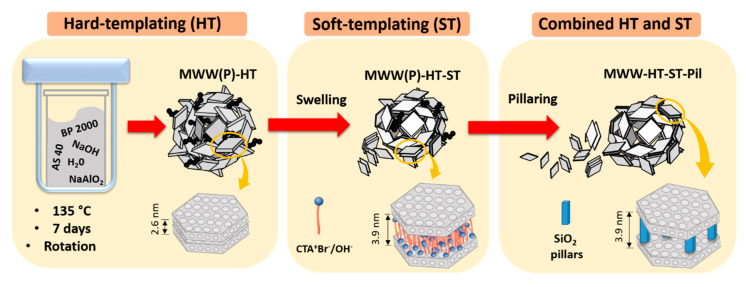
General scheme of the obtained material combining HT and ST approaches.

The surface areas (*S_BET_*) for standard-MWW, MWW-HT and MWW-HT-ST-Pil were estimated to be 636, 306 and 788 m^2^ g^−1^, respectively. The increase in *S_BET_* for MWW-HT-ST-Pil was a consequence of the pillaring procedure. The difference between the *S_BET_* values of MWW-HT and standard-MWW might have been a consequence of the carbon impurities present in the micropores/surface, which were not completely removed by calcination. The microporous volume (*V_micro_*) of standard-MWW, MWW-HT, and MWW-HT-ST-Pil was determined to be 0.14, 0.12 and 0.15 cm³ g^−1^, respectively. The decrease in *V_micro_* (compared with that of the standard-MWW zeolite) could be related to the dissolution of some MWW crystals during the swelling step and/or obstruction of micropores by silica during pillaring and the presence of carbon particles within the micropores. The total pore volume (*V_TP_*) of standard-MWW, MWW-HT, and MWW-HT-ST-Pil was estimated to be 0.78, 1.54 and 0.52 cm³ g^−1^, respectively. The interparticle pore volume (*V_int_*) was calculated by subtracting the *V_TP_* (obtained at a *p*/*p*^0^ value of 0.99) and the pore volume obtained at a *p*/*p*^0^ value of 0.6 (corresponding to pore sizes of 17–280 Å). MWW-HT-ST-Pil and MWW-HT showed *V_int_* values of 0.48 cm³ g^−1^ and 1.39 cm³ g^−1^, respectively, which indicated that *V_int_* was reduced by 55% after the ST procedure. The standard-MWW sample showed a *V_int_* value of 0.50 cm^3^ g^−1^, i.e., close to its *V_TP_*. Figure 5 summarizes of the obtained materials with combined HT and ST approaches.

## 3. Materials and Methods

The reactants were purchased from Sigma-Aldrich (tetraethyl orthosilicate, colloidal silica AS 40, sodium hydroxide 98%, hexamethyleneimine 98%, cetrimonium bromide ≥98%), Riedel-de-Haen (sodium aluminate, 40–45% Al_2_O_3_) and Cabot (carbon black BP 2000).

Synthesis with the HT method was performed using a procedure described in the literature with some modifications, i.e., at 135 °C and 7 days of crystallization using carbon black BP 2000 as a hard-template [30]. The theoretical molar gel composition was 1 SiO_2_:5 HMI:1 SiO_2_:0.019 Al_2_O_3_: 0.092 NaOH: 45 H_2_O, and the weight ratio between the BP 2000 and aluminum used for the synthesis was 0.083. The obtained LZP was named MWW(P)-HT. Synthesis of standard-MWW(P)—not using the HT and ST method—was also performed for comparison purposes. The materials were calcined for 12 h at 580 °C and named MWW-HT and MWW, respectively.

The ST approach was performed using the swelling approach described in the literature but without the use of TPA^+^ cations [33]. The precursor material was then swollen with hexadecyltrimethylammonium bromide with Br ions partially exchanged (50%) by OH ions; the final product was named MWW(P)-HT-ST. The pillaring was performed using tetraethyl orthosilicate and an MWW(P)-HT-ST weight ratio of 0.2. After calcination at 550 °C for 8 h, the material was named MWW-HT-ST-Pil.

The resulting materials were characterized by X-ray diffraction (XRD), sorption analysis using N_2_ as the probe molecule, atomic absorption spectroscopy (AAS), scanning electron microscopy (SEM) and transmission electron microscopy (TEM). The X-ray diffractograms were obtained using Bruker D2 Phaser equipment with a Lynxeye detector and Cu K-alpha radiation. N_2_ adsorption isotherms were obtained with a Micromeritics TriStar II 3020 system after degassing samples at 300 °C for 12 h. The surface area (*S_BET_*) was estimated by the Brunauer, Emmett and Teller (BET) method, while the micropore volume (*S_micro_*) was estimated by t-plot method. The total pore total volume (*V_TP_*) was estimated from the amount of N_2_ adsorbed at a relative pressure *p*/*p*^0^ = 0.99. The pore size distribution were estimated using the DFT method and a slit-like pore model. The morphology of the samples was studied by FE-SEM (field emission scanning electron microscopy) using a ZEISS Ultra-55 system with a working distance of 4.5 mm. The samples were also studied by electron microscopy using a Phillips (CM10 at 100 kV) microscope operating in transmission mode (TEM). The elemental analysis of silicon and aluminum was performed on a Perkin Elmer (model AAnalyst) with atomic absorption spectroscopy (AAS) equipment.

## 4. Conclusions

In this research work, we studied the physicochemical properties of MWW-type materials after combining HT, ST and pillaring methodologies. The obtained material showed intermediate properties from both approaches. The XRD pattern of the MWW-HT-ST-Pil material differed from standard-MWW and MWW-HT materials by the presence of basal reflections with a d001 spacing of 3.9 nm, indicating gallery heights of 1.4 nm. Moreover, the pore size distribution confirmed the presence of supermicropores and small mesopores formed by pillaring, and interparticle meso-/macro- pores obtained by the use of hard templates. SEM and TEM analyses showed that the ST approach led to the partial rupture of the microspherical particles formed by the HT approach, confirming that swelling procedures have an impact on the morphology of the final product. Therefore, it was possible to obtain a material with a combination of different pore system interparticle meso-/macro- pores, interlamellar mesopores and micropores, which may have potential for use in cascade-type reactions involving bulky reactants, the encapsulation of metal nanoparticles or as devices to support/trap biomolecules.
